# Low-Dose Indapamide vs. Hydrochlorothiazide in Idiopathic Hypercalciuria: A Randomized Prospective Trial

**DOI:** 10.3390/jcm14207426

**Published:** 2025-10-21

**Authors:** Maria Peraire, Jorge Guimerà, Antonia Costa-Bauzá, Pilar Sanchís, Felix Grases, Enrique Pieras

**Affiliations:** 1 Urology Department, Joan XXIII University Hospital, 43005 Tarragona, Spain; 2 Urology Department, Son Espases University Hospital, 07120 Palma de Mallorca, Spain; jorge.guimera@ssib.es (J.G.); enriquec.pieras@ssib.es (E.P.); 3 Nefro-Urologic Diseases Research Group, Fundación Instituto de Investigación Sanitaria Islas Baleares (IdISBa), 07122 Palma de Mallorca, Spain; 4 Renal Lithiasis and Pathological Calcification Group, Research Institute of Health Sciences (IUNICS), University of the Balearic Islands, 07122 Palma de Mallorca, Spain; antonia.costa@uib.es (A.C.-B.); pilar.sanchis@uib.es (P.S.); fgrases@uib.es (F.G.); 5 Department of Chemistry, University of the Balearic Islands, Health Research Institute of the Balearic Islands (IdISBa), 07122 Palma de Mallorca, Spain

**Keywords:** idiopathic hypercalciuria, indapamide, hydrochlorothiazide, treatment, kidney stones

## Abstract

**Background**: Idiopathic hypercalciuria (IH) is the most common genetic metabolic disorder in patients with urinary stones and is usually treated with thiazide-type diuretics. However, direct evidence comparing low-dose indapamide and hydrochlorothiazide is scarce. This study aimed to compare their safety, efficacy and adherence in patients with IH. **Methods**: In this randomized prospective trial, a total of 101 patients with IH were recruited at Son Espases University Hospital (2020–2023), assigned to receive indapamide 1.5 mg/day (n = 53) or hydrochlorothiazide 25 mg/day (n = 48), and followed for 18 months. Adverse events, biochemical parameters and therapeutic adherence were evaluated. **Results**: A total of 90.24% patients on indapamide and 85.71% on hydrochlorothiazide showed normal calciuria (*p* = 0.53). Both treatments increased serum urate (*p* = 0.62). Indapamide significantly reduced β-crosslaps (*p* < 0.05), suggesting bone protection. No significant differences were found in citraturia, uricosuria, phosphaturia, magnesiuria, magnesemia, natremia, or kalemia. Indapamide caused more mild adverse events, lowering adherence, while hydrochlorothiazide caused the only severe adverse effect—moderate hypokalemia. No differences in kidney stone recurrence were observed (*p* = 0.82). **Conclusions**: This is the first paper elucidating low-dose indapamide and hydrochlorothiazide efficacy and safety for the treatment of IH. No significant differences were observed between the two drugs in terms of adverse events or treatment adherence. A longer follow-up is needed to assess kidney stone recurrence.

## 1. Introduction

Idiopathic hypercalciuria (IH) is the most common metabolic disorder in patients with kidney stones, particularly in those with calcium stone formation [1]. This disorder, characterized by increased urinary calcium excretion (>250 mg/day in women, >300 mg/day in men, or >4 mg/kg body weight in both sexes) in the absence of hypercalcemia and other known causes of hypercalciuria [2,3], results from disrupted calcium transport in the intestine [1,4,5], kidney [6,7,8], and bone [9,10], influenced by genetic and dietary factors. IH affects 40–50% of stone formers, 35–40% of their close relatives, and 5–10% of the general population. Most stones are calcium oxalate or mixed calcium oxalate–phosphate, highlighting the dual impact of IH on kidney and skeletal health [11].

Management of patients with IH is crucial for preventing the recurrence of stone events. Reducing urinary supersaturation requires multiple interventions, including increased fluid intake, dietary counseling, and pharmacological therapy [12,13].

Thiazide diuretics remain the standard therapy for IH, with hydrochlorothiazide historically being the most widely studied. High doses (50–100 mg/day) are demonstrably effective at reducing urinary calcium and stone recurrence, but dose-related side effects limit their use. Indapamide, a thiazide-like sulfonamide, shares a similar mechanism of action and has demonstrated comparable efficacy in reducing urinary calcium excretion at lower doses (1.5–2.5 mg/day), with a more favorable tolerability profile and fewer reported adverse effects [14,15,16].

However, no randomized comparative studies have included a direct evaluation of the efficacy of these drugs at very low doses. Moreover, the incidence of adverse events differs between them. Given a comparable reduction in calciuria, their distinct side effect profiles may influence treatment selection in clinical practice. To address this gap, the present study is the first to evaluate differences in adverse events between low-dose indapamide (1.5 mg/day) and hydrochlorothiazide (25 mg/day) in the treatment of IH. Secondary endpoints include efficacy in reducing calciuria, changes in 24-h urinary metabolic profiles over 18 months, and treatment adherence, to help identify the most suitable option for IH management.

## 2. Materials and Methods

### 2.1. Trial Oversight and Design

The trial protocol was registered and received approval from the Research Committee of the Son Espases University Hospital (CI-374-20) and the Ethics Committee for Research of the Government of the Balearic Islands (CEI-IB) (IB 4273/20 EPA) prior to patient enrollment. The trial was carried out in compliance with all applicable regulations. All patients provided written informed consent prior to their participation.

As a post-authorization observational study involving two already approved drugs, registration in a public international registry (e.g., ClinicalTrials.gov or EU Clinical Trials Register) was not required.

### 2.2. Study Population

The study population was consecutively recruited from a stone referral center in Palma de Mallorca (Son Espases University Hospital) between December 2020 and December 2023. Key eligibility criteria included patients aged between 18 and 65 years old with a history of stone disease, idiopathic hypercalciuria (>250 mg/day for women, >300 mg/day for men), absence of hypercalcemia, normal levels of PTH, calcitriol, and phosphorus, and a 24-h blood and urine analysis available within the 6 months prior to the study inclusion.

The trial excluded pregnant or breastfeeding women, patients with active urinary tract infection, a glomerular filtration rate <60 mL/min/1.73 m^2^, low systolic or diastolic blood pressure, use of drugs known to influence calcium metabolism (loop diuretics, carbonic anhydrase inhibitors, xanthine oxidase inhibitors, NSAIDs, cyclosporine, corticosteroids, antihypertensive or antiarrhythmic treatment, vitamin D, calcium supplements, bisphosphonates, denosumab, potassium citrate or sodium bicarbonate), with known bone diseases (Paget’s disease, β-crosslaps > 0.8 ng/mL, and/or bone densitometry showing osteopenia or osteoporosis) and with specific diseases (hyperparathyroidism or hyperthyroidism, distal renal tubular acidosis (RTA), multiple myeloma, neoplasms, sarcoidosis, Cushing’s disease).

### 2.3. Sample Size Calculation

The sample size calculation was informed by previous studies evaluating the safety profile of indapamide in patients with idiopathic hypercalciuria and recurrent nephrolithiasis, which consistently reported low rates of treatment-limiting adverse events compared to thiazide diuretics [17,18]. Considering the variability described in these studies, we defined a 24% absolute difference in adverse event rates between groups as the minimum clinically meaningful effect size to be detected. Assuming a two-sided test, an alpha risk of 0.05, and a beta risk of 0.20, the calculation indicated that 40 patients per group would be required. Allowing for a 10% loss to follow-up, the final target sample size was adjusted accordingly.

### 2.4. Randomization, Treatment, Data Collection and Follow-Up

Eligible patients were scheduled for consultation with the principal investigator to discuss study participation, including benefits, potential adverse events, and follow-up methodology. A medical history summary, including prior imaging (CT scans and urological ultrasounds available within the 6 months prior to the study inclusion), and a physical examination were performed. Eligible patients meeting all inclusion criteria were consecutively enrolled and randomly assigned to treatment arms using computer-generated block randomization (blocks of 4) created in Excel. The randomization sequence was concealed until assignment, ensuring that the investigator could not predict treatment allocation in advance.

The study was open-label, with both patients and investigators aware of the assigned treatment. To minimize potential bias, clinical outcomes, adverse events, and treatment adherence were prospectively assessed at 3, 6, 12, and 18 months, with comprehensive clinical histories and laboratory evaluations systematically documented at each visit. A physical examination with vital sign measurement, along with 24-h urine (including urinary volume, 2 h urinary calcium, 2 h urinary citrate, 2 h urinary creatinine, 2 h urinary pH, 24 h urinary calcium, 24 h urinary citrate, 24 h urinary creatinine, 24 h urinary phosphate, 24 h urinary magnesium, 24 h urinary oxalate and 24 h urinary urate), blood tests (including creatinine, glucose, urate, urea, calcium, phosphate, sodium, potassium, magnesium, PTH, 25-hydroxyvitamin D, β-crosslaps, liver enzymes (AST, ALT, GGT), alkaline phosphatase, cholesterol (LDL, HDL, total cholesterol) and triglycerides), and urine cultures, were conducted. Patients were advised to consume ≥ 2 L of water daily and avoid salt in meals.

### 2.5. End-Points

The main trial objective was to investigate whether there are differences in adverse events between indapamide 1.5 mg/day and hydrochlorothiazide 25 mg/day in the treatment of patients with IH. Adverse events were defined as any sign (including alterations in laboratory variables), symptom, or undesirable or adverse disease occurring after or simultaneously with the use of the medication.

Secondary endpoints included comparing the therapeutic efficacy (reduction in calciuria) between both drugs, describing the evolution of the 24-h urine metabolic study over 18 months, and treatment adherence evaluation. The effect on blood pressure and stone events (pain, requirement for surgical intervention, need for urinary diversion, or medical treatment) was also observed.

### 2.6. Statistical Analysis

Quantitative variables were expressed as mean and standard deviation, while qualitative variables were expressed as proportion. Intragroup analysis was performed using the ANOVA test for repeated measures, with the LSD as a post hoc test to determine differences at different time points (0, 3, 6, 12, and 18 months) for quantitative variables. Baseline characteristics between groups were analyzed using Student’s T- test for independent samples or the Mann–Whitney U test for quantitative variables. Between-group analysis was conducted using the ANOVA test for repeated measures. For qualitative variables, the chi-square test or Fisher’s exact test was used. Statistical analyses were performed using SPSS 28.0 (SPSS Inc., Chicago, IL, USA). A *p*-value less than 0.05 was considered statistically significant.

## 3. Results

**Figure 1:**
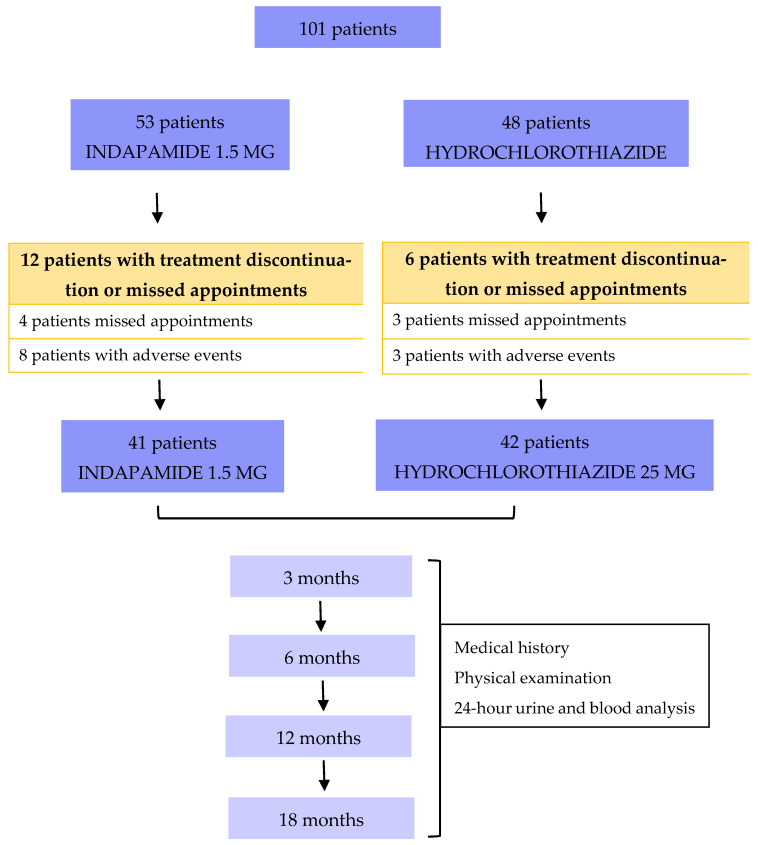
Study design, including enrolled patients and losses to follow-up due to adverse events or missed appointments.

A total of 101 patients matching the inclusion criteria were enrolled and randomized for the study, 53 and 48 in the indapamide group vs. hydrochlorothiazide group, respectively. Of those, 12 and 6 in the indapamide vs. hydrochlorothiazide treatment arm experienced adverse effects leading to treatment discontinuation or missed appointments (Figure 1), respectively. Therefore, 83 patients (41 in the indapamide group vs. 42 in the hydrochlorothiazide group) completed the 18-month follow-up.

There was no statistically significant difference in baseline characteristics between the 101 patients included in the trial and randomized in both groups (Table 1). Overall, 57 (56.4%) were male and the mean age was 46 years (range 20–65, SD 10 years). Both groups had a mean BMI of 25.7 kg/m^2^ (SD 4.1) and a mean daily water intake of 1.7 L (SD 0.48). Smoking prevalence was 34% in the indapamide group and 29.2% in the hydrochlorothiazide group. No significant comorbidity differences were found between groups (diabetes, dyslipidemia, obesity, or HIV).

Regarding lithiasis history, both groups had a mean onset age of 32 years (SD 10). A family history of lithiasis was reported by 64.2% of indapamide patients and 47.9% of hydrochlorothiazide patients, with an average of one affected first-degree relative (SD 1.1) in both groups. At treatment initiation, 73.6% of the indapamide group and 83.3% of the hydrochlorothiazide group exhibited lithiasis in imaging studies conducted within six months prior to study inclusion, with a mean stone size of 5.5 mm (SD 2.6) in both groups. Lithiasis characteristics are presented in Table 2.

### 3.1. Primary End Point

Of the 53 indapamide and 48 hydrochlorothiazide patients, 12 and 6 did not complete follow-up, respectively. In the indapamide group, four patients (7.5%) missed visits, and eight (15%) discontinued treatment due to minor adverse events (pruritus, dizziness, polyuria, discomfort). In the hydrochlorothiazide group, three patients (6.2%) missed visits, and two (4.2%) discontinued for similar reasons. One extra patient (2.1%) in the hydrochlorothiazide group experienced a serious adverse event—moderate hypokalemia (2.6 mEq/L) with ECG changes—resulting in drug discontinuation.

Among the 83 patients completing 18 months of follow-up (41 indapamide group vs. 42 hydrochlorothiazide group), four experienced adverse events. In the indapamide group, two patients (4.8%) experienced adverse events: one with constipation and one with hyperuricemia (>8 mg/dL). In the hydrochlorothiazide group, two patients (4.7%) presented with persistent hypertriglyceridemia (>200 mg/dL). There were no significant group differences in terms of adverse events or follow-up losses (Table 3).

### 3.2. Secondary End Points

**Figure 2:**
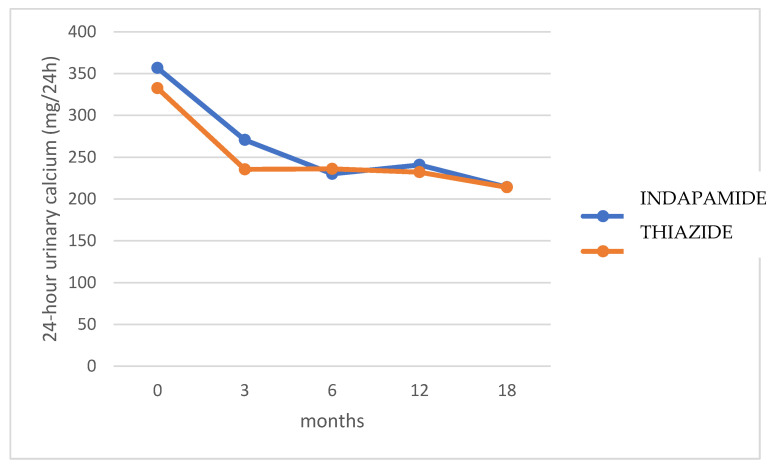
Evolution of 24-h urinary calciuria in patients treated with hydrochlorothiazide and indapamide. Normal reference ranges for 24-h urinary calcium: <250 mg/day in women and <300 mg/day in men.

**Figure 3:**
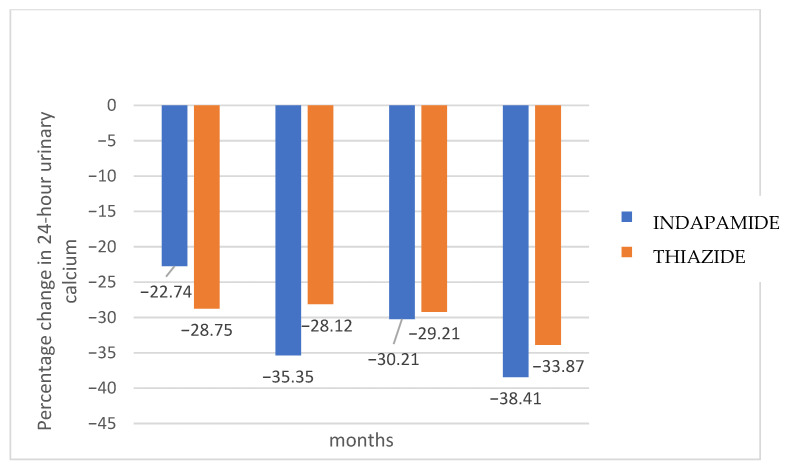
Percentage change of calcium in 24-h urine for each treatment group relative to the mean baseline values of the studied population.

No significant differences were found in 24-h urinary calcium excretion between both treatment groups at baseline and 3, 6, 12, and 18 months (*p* = 0.368) (Figure 2). Figure 3 shows the percentage change in 24-h urinary calcium for each treatment group compared to baseline values. Hydrochlorothiazide presented a faster effect in lowering calcium excretion, maintaining it at normal levels from the third month, while indapamide required 6 months. After 18 months, indapamide treatment resulted in a mean decrease of 38.4% (SD 19.8), while hydrochlorothiazide decreased urinary calcium by 33.8% (SD 20.2) (*p* = 0.145). However, a higher proportion of indapamide relative to hydrochlorothiazide group patients (90.2 vs. 85.7%) maintained normal urinary calcium levels at 18 months, although without statistical significance (*p* = 0.526).

No significant differences were found in urine volume, citraturia, uricosuria, phosphaturia, magnesiuria, magnesemia, natremia, or kalemia (all *p* > 0.05). Both treatments increased serum urate, with no difference between them (*p* = 0.618). Indapamide significantly reduced β-crosslaps (*p* = 0.049). The graphs and tables depicting the evolution of the parameters from the metabolic blood and urine study can be found in Supplementary Materials.

After excluding the 18 patients with missed appointments or adverse events leading to treatment discontinuation at the beginning of the trial from follow-up, adherence was confirmed for all 83 patients who completed the study, based on repeated clinical interviews and pharmacy data. Despite four adverse events during follow-up, none led to treatment discontinuation. In conclusion, 77.4% (41/53) of patients in the indapamide group vs. 87.6% (42/48) in the hydrochlorothiazide group displayed good therapeutic adherence, with no significant differences between groups (*p* = 0.184).

Significant differences were found in the evolution of systolic blood pressure (SBP) (*p* = 0.049) and diastolic blood pressure (DBP) (*p* < 0.001) in patients treated with indapamide, as well as in the evolution of SBP (*p* < 0.001) and DBP (*p* < 0.001) in patients treated with hydrochlorothiazide. However, no differences were observed in the comparison between the two treatment groups (SBP *p* = 0.452; DBP *p* = 0.785), with mean blood pressure remaining within normal limits throughout.

Of the 83 patients with complete follow-up, 15 experienced lithiasis events (7 in the indapamide group vs. 8 in the hydrochlorothiazide group) with no significant differences between groups (*p* = 0.815). Seven patients (8.4%) had painless expulsions, and eight patients (9.6%) had mild discomfort. Most patients experienced one event, and no surgeries were needed, with all cases treated conservatively. There was a median of two pre-treatment lithiasis events and one during treatment.

## 4. Discussion

In this randomized prospective trial, we aimed to compare the adverse event profiles of low-dose indapamide 1.5 mg and hydrochlorothiazide 25 mg in treating 101 patients with IH and to evaluate their long-term efficacy and adherence.

Adverse events with 25 mg hydrochlorothiazide are infrequent (<10%) and generally dose-dependent, minimized by using the lowest effective dose, including hypokalemia, hyponatremia, hypomagnesemia, hyperuricemia, and skin rashes. Indapamide 1.5 mg caused fewer adverse events, with hypokalemia being the most common.

In our study, 18.8% of patients in the indapamide group and 10.4% in the hydrochlorothiazide group experienced drug-related adverse events, with no significant difference between groups. Indapamide was associated with more adverse events and treatment discontinuations, whereas hydrochlorothiazide was the only drug to cause a severe adverse event (hypokalemia with ECG changes) leading to discontinuation.

To the best of our knowledge, no study has prospectively evaluated and compared the impact of very-low-dose indapamide and hydrochlorothiazide in the treatment of patients with IH. Only two historical studies with short follow-up periods (Lemieux G et al., 1986 [18]; and Martins MC et al., 1996 [15]) assessed the effects of standard-dose indapamide and hydrochlorothiazide in this setting.

Lemieux G et al. (1986) evaluated indapamide 2.5 mg/day for 3 months in 26 IH patients, showing a 52% reduction in urinary calcium excretion (*p* < 0.05), which reverted to baseline after discontinuation. This reduction was similar to that observed with hydrochlorothiazide 100 mg/day, which reduced calciuria by 50% [18]. Martins MC et al. (1996) compared hydrochlorothiazide 50 mg/day vs. indapamide 2.5 mg/day in 12 IH patients over 3 months, observing significant reductions in calciuria (*p* < 0.005) with no difference between the drugs. Comparing different hydrochlorothiazide doses (50 mg/day, 25 mg/day, 12.5 mg/day) showed a significant decrease with 50 mg/day (*p* < 0.001), similar to indapamide, and with 25 mg/day (*p* < 0.05), though smaller. No significant reduction was seen with 12.5 mg/day [15].

In a retrospective review, da Silva Cunha et al. (2020) demonstrated that thiazide and thiazide-like diuretics reduce urinary calcium excretion and lower stone recurrence in hypercalciuric patients, with potential benefits for bone metabolism. Their cohort included 28 individuals treated with hydrochlorothiazide (25 mg/day) or indapamide (2.5 mg/day) for at least one year, with a metabolic study performed at 3 months after treatment initiation. Nevertheless, the authors underscored the absence of prospective head-to-head trials directly comparing these agents [19].

On the other hand, Alonso et al. (2012) demonstrated that indapamide at a dose of 1.5 mg/day significantly reduced urinary calcium excretion while maintaining good tolerability [20]. These findings support the effectiveness of lower doses of indapamide and reinforce the rationale for minimizing drug exposure to reduce the risk of dose-related adverse effects.

Based on these data, we selected the lowest evidence-based effective doses—indapamide 1.5 mg/day and hydrochlorothiazide 25 mg/day—to balance efficacy with a lower risk of adverse effects. To our knowledge, this is the first prospective study to directly compare these low-dose regimens over an extended follow-up period in the treatment of IH.

In the present study, both drugs were potent inhibitors of urinary calcium excretion, with no significant differences between groups. Hydrochlorothiazide, however, exhibited a faster effect, lowering calcium excretion to normal levels by the third month, whereas indapamide required six months of use to achieve the same effect. Therefore, hydrochlorothiazide may be particularly beneficial for patients with highly recurrent nephrolithiasis, where rapid control of hypercalciuria is required. Conversely, a higher proportion of indapamide-treated patients achieved normal calciuria at 18 months.

No significant differences were found between indapamide 1.5 mg/day and hydrochlorothiazide 25 mg/day in the evolution of citraturia, uricosuria, phosphaturia, or magnesiuria over 18 months. Neither drug affected blood glucose or renal function. This aligns with the findings of Singh et al. who reported that patients with kidney stones receiving thiazide therapy exclusively for stone prophylaxis did not exhibit an increased risk of developing diabetes mellitus [21]. Both drugs increased serum urate levels without significant differences. No major changes were observed in magnesium, sodium, or potassium concentrations throughout the follow-up, remaining their mean values within the normal range in both treatment groups. However, one patient receiving hydrochlorothiazide developed moderate hypokalemia leading to discontinuation. This isolated case is consistent with the well-known kaliuretic effect of hydrochlorothiazide, resulting from enhanced sodium delivery to the distal nephron and subsequent potassium exchange under aldosterone influence. Despite this event, the overall potassium profile did not differ significantly between groups, suggesting that, at the administered doses, both treatments were generally safe regarding electrolyte balance. Both treatments lowered LDL cholesterol (more significantly with hydrochlorothiazide, which could make this drug the preferred choice for patients with multiple cardiovascular risk factors) and slightly increased triglycerides. Indapamide showed potential bone protection, indicated by a significant decrease in β-crosslaps, a finding that warrants further long-term investigation and could influence our choice of medication for patients with hypercalciuria linked to excessive bone turnover. Consistently, the literature suggests that both hydrochlorothiazide and indapamide can reduce fracture risk by decreasing urinary calcium excretion and improving overall calcium balance [22,23]. However, the effects of thiazide and thiazide-like diuretics on bone may extend beyond their anti-calciuric properties. In vitro studies suggest that these drugs can stimulate osteoblastic bone formation by increasing osteocalcin production and upregulating the thiazide-sensitive sodium-chloride co-transporter, which promotes osteoblast proliferation [24]. Additionally, thiazides have been shown to inhibit osteoclastic bone resorption, an effect that may help maintain bone mass [25]. Thus, thiazide and thiazide-like therapy is recommended for bone mass and strength improvement in hypercalciuric patients.

Regarding treatment adherence, we observed a higher treatment discontinuation due to adverse events in the indapamide group at baseline (15 vs. 6.2% for hydrochlorothiazide) and a greater overall discontinuation when including patients who missed follow-up without specified reasons (22.5 vs. 12.4% for hydrochlorothiazide). While not statistically significant, this trend suggests that real-world patient tolerance may favor hydrochlorothiazide in some cases. The relatively high dropout rate, particularly in the indapamide group, should be considered as it may bias the assessment of real-world efficacy and safety.

Regarding stone events, several studies have shown the efficacy of thiazide diuretics in reducing urinary calcium excretion and associated lithiasis events [13,26]. As mentioned earlier, lower doses of these drugs have similar effects on calciuria while reducing the risk of adverse events. However, it is unclear whether low doses of hydrochlorothiazide or indapamide have the same impact on preventing lithiasis events as higher doses. A 2018 study comparing high vs. low doses of hydrochlorothiazide and indapamide found no differences in lithiasis risk [27]. More recently, Dhayat NA et al. evaluated the efficacy of standard (50 mg) vs. low doses (25 mg and 12.5 mg) of hydrochlorothiazide in preventing recurrent calcium stones over 3 years, finding no relationship between dose and recurrence, also compared with placebo [28]. However, only 63% of the population had hypercalciuria, which differs from our study and may account for differences in lithiasis recurrence, potentially identifiable with longer follow-up. Additionally, the study did not specify the type of stones (COM, COD, or mixed), which may explain the lack of clinical outcomes from hydrochlorothiazide treatment. In our trial no statistically significant differences in stone events were observed between the two treatments over 18 months. A longer follow-up may reveal differences in event frequency between the drugs.

Our study has some limitations that should be considered when interpreting the results. First, it is a single-center study with a relatively small sample size, and statistical power considerations might be warranted. The sample size was calculated to detect clinically meaningful differences in adverse-event rates—our primary endpoint—so the study may have been underpowered to identify smaller but potentially relevant differences in secondary outcomes such as calciuria reduction, stone recurrence, metabolic parameters, and treatment adherence. Consequently, the lack of statistically significant differences in these domains should not be interpreted as evidence of equivalence between treatments, as a potential Type II error cannot be excluded. Larger, adequately powered multicenter trials designed to evaluate these outcomes are needed to determine whether the nonsignificant results observed here reflect true similarity or insufficient power. Second, although follow-up was sufficient to assess changes in calciuria, metabolic parameters, adverse events, and treatment adherence, a longer follow-up is needed to more rigorously evaluate lithiasis recurrence. Third, adverse events were self-reported, lacking medical evaluation, which may have introduced reporting bias. Fourth, dietary intake was uncontrolled and could have confounded metabolic outcomes and treatment effectiveness. Fifth, the absence of a placebo control group limits the ability to assess treatment effects against no intervention; however, the study was designed as a head-to-head comparison of two active standard-of-care treatments to provide clinically relevant information, as a placebo group would have been ethically unacceptable. Finally, patient dropout and missed appointments may have introduced bias, as participants lost to follow-up could differ from those who completed the study, potentially affecting the interpretation and generalizability of the results.

Taken together, these limitations should be considered when interpreting our findings, particularly regarding their generalizability and the interpretation of non-significant results. Nevertheless, our study provides valuable insights into the comparative use of indapamide and hydrochlorothiazide in real-world settings. Our results suggest that indapamide, despite its favorable reputation, may present a higher discontinuation rate, underscoring the importance of a patient-centered approach that considers individual metabolic profiles and comorbidities when selecting therapy. To strengthen these observations and draw definitive conclusions on lithiasis recurrence as well as long-term metabolic and bone-related effects, future research should prioritize larger multicenter trials with extended follow-up.

## 5. Conclusions

This is the first paper to report that low-dose indapamide and hydrochlorothiazide are effective and safe for lowering urinary calcium in IH, with no significant differences in terms of adverse events or adherence.

Hydrochlorothiazide showed a trend toward a faster reduction in urinary calcium, which could be advantageous for patients with recurrent stones, whereas indapamide appeared to be associated with higher long-term rates of calciuria normalization and a possible bone-protective effect. Taken together, our findings suggest that either drug can be considered a first-line option for IH at low doses to balance efficacy and safety.

Future studies with longer follow-up are warranted to evaluate kidney stone recurrence, bone metabolism and predictors of individual response.

## Figures and Tables

**Table 1:** Sociodemographic variables of the study groups.

	Indapamide (n = 53)	Hydrochlorothiazide (n = 48)	*p*
Age (years)	46 ± 10	46 ± 10	0.176
Gender (female)	22 (41.5%)	22 (45.8%)	0.826
BMI ^1^ (kg/m^2^)	25.7 ± 4.1	25.7 ± 4.1	0.233
Water intake (liters)	1.7 ± 0.48	1.7 ± 0.48	0.306
Smokers	18 (34%)	14 (29.2%)	0.641
**Personal history**			
Type 2 Diabetes Mellitus	3 (5.7%)	1 (2.1%)	0.616
Dyslipidemia	6 (11.3%)	7 (14.6%)	1.000
Obesity	5 (9.4%)	8 (16.7%)	0.520
HIV	3 (5.7%)	0	0.494

^1^ BMI: Body Mass Index.

**Table 2:** Characteristics and urolithiasis history of the study groups.

	Indapamide (n = 53)	Thiazide (n = 48)	*p*
Age at onset of urolithiasis	32 ± 10	32 ± 10	0.603
Patients with a family history of urolithiasis	34 (64.2%)	23 (47.9%)	0.175
Number of first-degree affected relatives	1.0 ± 1.1	1.0 ± 1.1	0.411
Patients with urolithiasis at treatment initiation	39 (73.6%)	40 (83.3%)	0.443
Largest stone size	5.5 ± 2.6	5.5 ± 2.6	0.968
**Laterality**			0.034
Right	12 (22.6%)	7 (14.6%)	
Left	14 (26.4%)	28 (58.3%)	
Bilateral	27 (50.9%)	13 (27%)	
**Number**			0.130
Single	16 (30.2%)	24 (50%)	
Multiple	37 (69.8%)	24 (50%)	
**Urinary stone composition**			0.229
Calcium oxalate dihydrate + hydroxyapatite	14 (26.4%)	4 (8.3%)	
Calcium oxalate dihydrate	9 (17%)	7 (14.6%)	
Calcium oxalate monohydrate	9 (17%)	4 (8.3%)	
Calcium oxalate dihydrate + monohydrate	7 (13.2%)	6 (12.5%)	
Calcium oxalate monohydrate + dihydrate	4 (7.5%)	0	
Hydroxyapatite	5 (9.4%)	0	
Brushite + hydroxyapatite + COD	0	3 (6.3%)	
Brushite + hydroxyapatite	0	1 (2.1%)	

**Table 3:** Adverse events and missed appointments observed in both treatment groups.

Indapamide (n = 53)		Hydrochlorothiazide (n = 48)		
Number of patients with adverse events, %		Number of patients with adverse events, %		*p*
At treatment initiation	**8 (15%)** 2 pruritus 3 dizziness 1 polyuria 2 discomfort	At treatment initiation	**3 (6.2%)** 1 pruritus 1 discomfort 1 hypokalemia	0.154
During follow-up	**2 (3.8%)** 1 constipation 1 hyperuricemia	During follow-up	**2 (4.2%)** 2 hypertriglyceridemia	0.919
Total	**10 (18.8%)**	Total	**5 (10.4%)**	0.233
Missed appointments with indapamide		Missed appointments with hydrochlorothiazide		
	4 (7.5%)		3 (6.2%)	0.798

## Data Availability

The original contributions presented in this study are included in the article/supplementary material. Further inquiries can be directed to the corresponding author.

## Supplementary Materials

The following supporting information can be downloaded at: https://www.mdpi.com/article/10.3390/jcm14207426/s1. Figure S1. a: Evolution of the mean 24-h urine volume in patients treated with hydrochlorothiazide and indapamide; b: evolution of the mean 24-h urine citraturia in patients treated with hydrochlorothiazide and indapamide; c: evolution of the mean 24-h urine uricosuria in patients treated with hydrochlorothiazide and indapamide; d: evolution of the mean 24-h urine phosphaturia in patients treated with hydrochlorothiazide and indapamide; e: evolution of the mean 24-h urine magnesiuria in patients treated with hydrochlorothiazide and indapamide; f: evolution of the mean serum magnesium in patients treated with hydrochlorothiazide and indapamide; g: evolution of the mean serum sodium in patients treated with hydrochlorothiazide and indapamide; h: evolution of the mean serum potassium in patients treated with hydrochlorothiazide and indapamide; i: evolution of the mean serum urate in patients treated with hydrochlorothiazide and indapamide. Figure S2. a: Evolution of the mean serum β-crosslaps in patients treated with hydrochlorothiazide and indapamide; b: evolution of the mean serum calcifediol in patients treated with hydrochlorothiazide and indapamide; c: evolution of the mean serum parathyroid hormone in patients treated with hydrochlorothiazide and indapamide; d: evolution of the mean blood glucose in patients treated with hydrochlorothiazide and indapamide; e: evolution of the mean serum creatinine in patients treated with hydrochlorothiazide and indapamide; f: evolution of the mean serum calcium in patients treated with hydrochlorothiazide and indapamide; g: evolution of the mean serum LDL cholesterol in patients treated with hydrochlorothiazide and indapamide; h: evolution of the mean serum HDL cholesterol in patients treated with hydrochlorothiazide and indapamide; I: evolution of the mean serum triglycerides in patients treated with hydrochlorothiazide and indapamide. Table S1. Intragroup comparisons of patients treated with indapamide. a: *p* < 0.05 vs. corresponding pretreatment value; b: *p* < 0.05 vs. corresponding 3-month value; c: *p* < 0.05 vs. corresponding 6-month value; d: *p* < 0.05 vs. corresponding 12-month value. Table S2. Intragroup comparisons of patients treated with hydrochlorothiazide. a: *p* < 0.05 vs. corresponding pretreatment value; b: *p* < 0.05 vs. corresponding 3-month value; c: *p* < 0.05 vs. corresponding 6-month value; d: *p* < 0.05 vs. corresponding 12-month value. Table S3. Intergroup comparisons of patients treated with indapamide vs. hydrochlorothiazide. *: months in which the difference between treatment groups was <0.05.

## Abbreviations

The following abbreviations are used in this manuscript:

IH	Idiopathic hypercalciuria
BMI	Body Mass Index
SD	Standard Deviation
COD	Calcium Oxalate Dihydrate
COM	Calcium Oxalate Monohydrate
