# Supplementary material for: Low-Dose Indapamide vs. Hydrochlorothiazide in Idiopathic Hypercalciuria: A Randomized Prospective Trial
Source: J Clin Med. 2025 Oct 21;14(20):7426. doi: 10.3390/jcm14207426 (PMC12564931; doi:10.3390/jcm14207426)
Supplement: Supplementary file 1 [file jcm-14-07426-s001.zip › jcm-3878306-supplementary.pdf]

Supplementary Materials

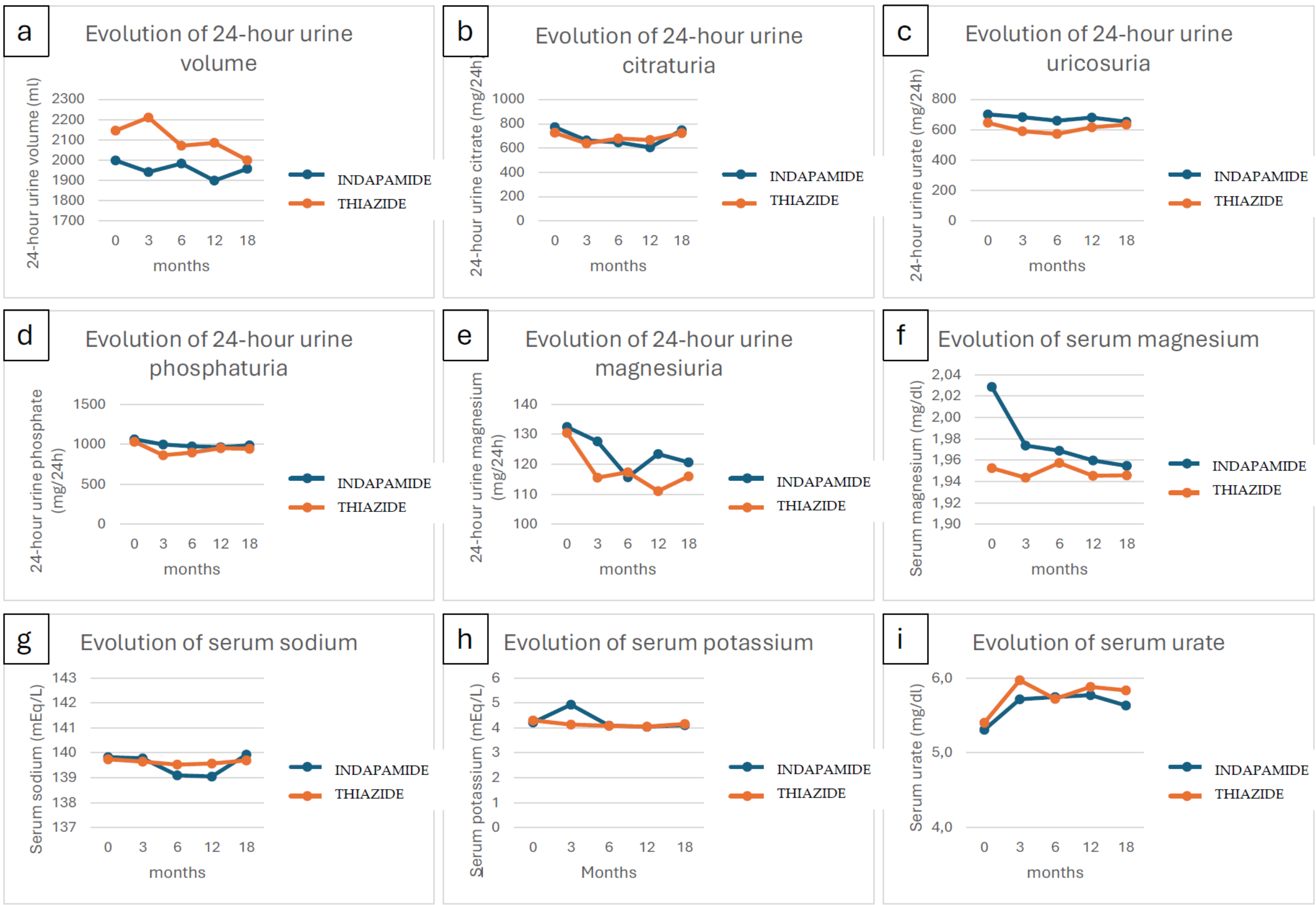

Figure S1. a: Evolution of the mean 24-hour urine volume in patients treated with hydrochlorothiazide and indapamide; b: evolution of the mean 24-hour urine citraturia in patients treated with hydrochlorothiazide and indapamide; c: evolution of the mean 24-hour urine uricosuria in patients treated with hydrochlorothiazide and indapamide; d: evolution of the mean 24-hour urine phosphaturia in patients treated with hydrochlorothiazide and indapamide; e: evolution of the mean 24-hour urine magnesiuria in patients treated with hydrochlorothiazide and indapamide; f: evolution of the mean serum magnesium in patients treated with hydrochlorothiazide and indapamide; g: evolution of the mean serum sodium in patients treated with hydrochlorothiazide and indapamide; h: evolution of the mean serum potassium in patients treated with hydrochlorothiazide and indapamide; i: evolution of the mean serum urate in patients treated with hydrochlorothiazide and indapamide.

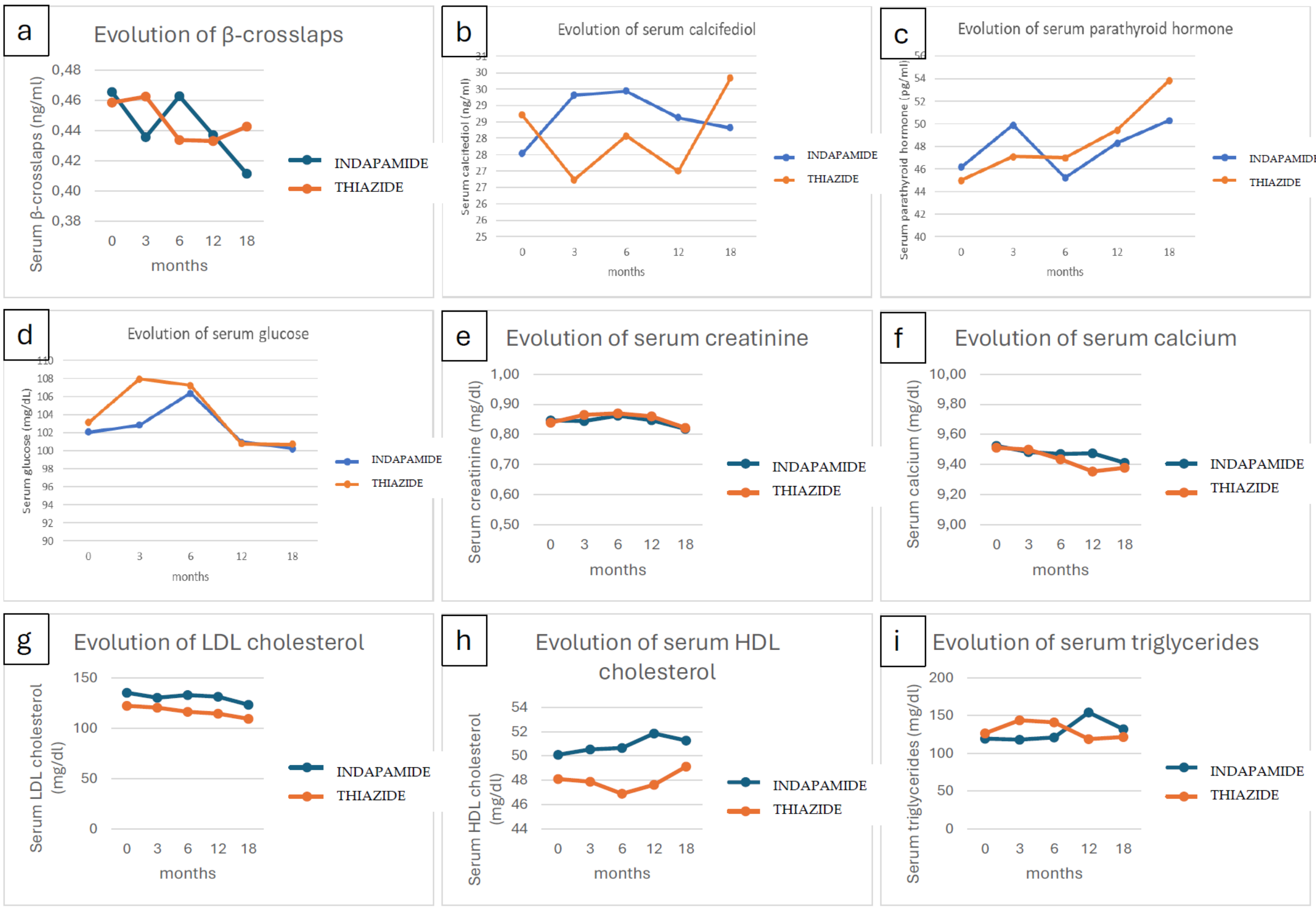

Figure S2. a: Evolution of the mean serum β-crosslaps in patients treated with hydrochlorothiazide and indapamide; b: evolution of the mean serum calcifediol in patients treated with hydrochlorothiazide and indapamide; c: evolution of the mean serum parathyroid hormone in patients treated with hydrochlorothiazide and indapamide; d: evolution of the mean blood glucose in patients treated with hydrochlorothiazide and indapamide; e: evolution of the mean serum creatinine in patients treated with hydrochlorothiazide and indapamide; f: evolution of the mean serum calcium in patients treated with hydrochlorothiazide and indapamide; g: evolution of the mean serum LDL cholesterol in patients treated with hydrochlorothiazide and indapamide; h: evolution of the mean serum HDL cholesterol in patients treated with hydrochlorothiazide and indapamide; I: evolution of the mean serum triglycerides in patients treated with hydrochlorothiazide and indapamide.

	Indapamide (n = 41)															**ANOVA for repeated measures**
	**PRE**			**3 months**			**6 months**			**12 months**			**18 months**			
	Mean	±	SD	Mean	±	SD	Mean	±	SD	Mean	±	SD	Mean	±	SD	*p*
2h Urine volume (ml)	87	±	30	80	±	36	78	±	42	79	±	20	82	±	29	0,548
2h Urine calcium (mg/L)	18,3	±	7,7	15,2	±	9,4	17,4	±	18,9	14,8	±	9,2	15,0	±	8,9	0,355
2h Urine citrate(mg/L)	388	±	200	473	±	270	456	±	245	438	±	226	431	±	219	0,117
2h Urine creatinine (mg/dl)	115	±	37	136	±	61[a]	154	±	88[a]	128	±	58[c]	127	±	55[c]	**0,013**
2h Urine pH	5,8	±	0,5	6,0	±	0,7	6,0	±	0,7	6,0	±	0,7	6,0	±	0,7	0,380
24h Urine volume (ml)	1999	±	578	1941	±	605	1983	±	682	1899	±	702	1958	±	671	0,822
24h Urine calcium (mg/24h)	357	±	82	271	±	122[a]	230	±	107[a]	241	±	115[a]	214	±	66[a,b]	**<0,001**
24h Urine citrate (mg/24h)	772	±	914	664	±	436	647	±	339	605	±	252	746	±	368	0,399
24hUrine creatinine (mg/24h)	1610	±	425	1534	±	568	1490	±	475[a]	1496	±	482[a]	1493	±	506[a]	**0,044**
24hUrinePhosphte (mg/24h)	1062	±	241	996	±	342	974	±	302	962	±	316	985	±	317	0,326
24hUrineMagnesiu(mg/24h)	132	±	48	128	±	50	116	±	41	123	±	60	121	±	43	0,130
24h Urine oxalate (mg/24h)	34	±	18	28	±	13	27	±	12	31	±	11	37	±	19	0,254
24h Urine urate (mg/24h)	702	±	199	683	±	218	660	±	209	680	±	191	653	±	174	0,371
Serum glucose (mg/dl)	102	±	22	103	±	23	106	±	36	101	±	32	100	±	27	0,143
Serum urea (mg/dl)	35,0	±	6,8	35,9	±	7,9	34,3	±	7,2	34,2	±	8,0	33,9	±	8,9	0,453
Serum creatinine (mg/dl)	0,85	±	0,19	0,84	±	0,17	0,86	±	0,18	0,85	±	0,17	0,82	±	0,15[a,c,d]	**0,032**
Serum urate (mg/dl)	5,3	±	1,2	5,7	±	1,2[a]	5,7	±	1,3[a]	5,8	±	1,2[a]	5,6	±	1,3[a]	**0,003**
Serum calcium (mg/dl)	9,52	±	0,40	9,48	±	0,39	9,47	±	0,38	9,48	±	0,38	9,41	±	0,41	0,558
Serum phosphate (mg/dl)	3,47	±	0,57	3,25	±	0,55[a]	3,15	±	0,57[a]	3,15	±	0,51[a]	3,19	±	0,56[a]	**<0,001**
Serum magnesium (mg/dl)	2,03	±	0,18	1,97	±	0,12[a]	1,97	±	0,15[a]	1,96	±	0,16[a]	1,95	±	0,16[a]	**0,003**
Serum sodium (mEq/L)	139,8	±	2,0	139,8	±	1,9	139,1	±	2,3	139,0	±	2,1[a,b]	139,9	±	1,9[d]	**0,024**
Serum potassium (mEq/L)	4,22	±	0,40	4,93	±	0,79	4,09	±	0,40	4,04	±	0,39	4,11	±	0,30	0,369
LDL (mg/dl)	135	±	40	130	±	31	133	±	34	131	±	34	123	±	37	0,083
HDL (mg/dl)	50	±	10	51	±	10	51	±	12	52	±	11	51	±	9	0,560
Total cholesterol (mg/dl)	208	±	48	200	±	41	210	±	48	213	±	38	200	±	44	0,059
Triglycerides (mg/dl)	119	±	58	118	±	60	121	±	66	154	±	170	132	±	133	0,229
GOT (U/L)	20,6	±	5,4	21,3	±	7,0	20,1	±	4,8	20,7	±	6,5	20,1	±	4,6	0,735
GPT (U/L)	20	±	9	22	±	14	21	±	9	21	±	10	22	±	9	0,704
GGT (U/L)	29	±	24	29	±	28	27	±	19	26	±	12	28	±	17	0,485
Alkaline phosphatase (U/L)	74	±	23	73	±	24	72	±	23	73	±	24	73	±	23	0,868
PTH (pg/ml)	46	±	19	50	±	23	45	±	16	48	±	19	50	±	22	0,281
Calcitriol 1.25 D (ng/ml)	52	±	14	55	±	18	56	±	11	56	±	12	54	±	9	0,374
25 hydroxyvitamin D (ng/ml)	28	±	11	29	±	12	29	±	11	29	±	14	28	±	10	0,651
β-crosslaps (ng/ml)	0,47	±	0,21	0,44	±	0,21	0,46	±	0,23	0,44	±	0,21	0,41	±	0,20[a,c,d]	**0,049**
Systolic BP (mmHg)	127,6	±	7,6	127,2	±	6,4	126,3	±	5,6	125,3	±	3,7	124,7	±	4,1[a,b]	**0,049**
Diastolic BP (mmHg)	67,0	±	5,9	65,9	±	5,6	65,1	±	4,4[a]	63,6	±	4,4[a,b,c]	63,2	±	3,5[a,b,c]	**<0,001**

Table S1. Intragroup comparisons of patients treated with indapamide. *a: p<0.05 vs corresponding pretreatment value; b: p<0.05 vs corresponding 3-month value; c: p<0.05 vs corresponding 6-month value; d: p<0.05 vs corresponding 12-month value.* BP: blood pressure.

	Thiazide (n = 42)															**ANOVA for repeated measures**
	**PRE**			**3 months**			**6 months**			**12 months**			**18 months**			
	Mean	±	SD	Mean	±	SD	Mean	±	SD	Mean	±	SD	Mean	±	SD	*p*
2h Urine volume (ml)	75	±	28	66	±	23	61	±	29[a]	64	±	25[a]	67	±	19	**0,028**
2h Urine calcium (mg/L)	16,1	±	6,9	17,6	±	9,9	15,8	±	8,4	15,9	±	9,4	15,6	±	8,5	0,672
2h Urine citrate(mg/L)	482	±	290	623	±	435[a]	683	±	417[a]	656	±	406[a]	635	±	358[a]	**0,002**
2h Urine creatinine (mg/dl)	175	±	338	164	±	56	179	±	77	162	±	60	153	±	63	0,686
2h Urine pH	6,0	±	0,7	5,9	±	0,6	5,9	±	0,6	6,1	±	0,7	5,9	±	0,7	0,356
24h Urine volume (ml)	2146	±	765	2211	±	799	2071	±	712	2086	±	796	2000	±	635	0,238
24h Urine calcium (mg/24h)	333	±	61	235	±	90[a]	236	±	84[a]	232	±	86[a]	214	±	58[a]	**<0,001**
24h Urine citrate (mg/24h)	725	±	352	638	±	378	678	±	345	666	±	364	722	±	372	0,222
24hUrine creatinine (mg/24h)	1567	±	483	1411	±	435[a]	1415	±	399[a]	1453	±	418	1422	±	364[a]	**0,023**
24hUrinePhosphte (mg/24h)	1031	±	289	862	±	286[a]	895	±	315[a]	949	±	272[b]	943	±	273	**0,007**
24hUrineMagnesiu(mg/24h)	130	±	55	116	±	41[a]	117	±	37	111	±	38[a]	116	±	38[a]	**0,033**
24h Urine oxalate (mg/24h)	34	±	15	35	±	22	30	±	11	34	±	6	24	±	15	-
24h Urine urate (mg/24h)	646	±	201	590	±	178	573	±	161	616	±	179	634	±	195	0,058
Serum glucose (mg/dl)	103	±	23	108	±	41	107	±	49	101	±	28	101	±	33	0,342
Serum urea (mg/dl)	30,5	±	6,6	31,8	±	7,4	31,4	±	7,4	32,7	±	8,8	30,8	±	7,8	0,178
Serum creatinine (mg/dl)	0,84	±	0,19	0,87	±	0,18	0,87	±	0,18	0,86	±	0,19	0,82	±	0,17	0,327
Serum urate (mg/dl)	5,4	±	1,3	6,0	±	1,4[a]	5,7	±	1,2[a]	5,9	±	1,4[a]	5,8	±	1,3[a]	**0,001**
Serum calcium (mg/dl)	9,51	±	0,44	9,50	±	0,47	9,44	±	0,44	9,35	±	0,44	9,38	±	0,41	0,062
Serum phosphate (mg/dl)	3,25	±	0,47	3,25	±	0,68	3,13	±	0,67	3,09	±	0,61	3,22	±	0,62	0,178
Serum magnesium (mg/dl)	1,95	±	0,13	1,94	±	0,16	1,96	±	0,20	1,95	±	0,17	1,95	±	0,16	0,914
Serum sodium (mEq/L)	139,7	±	1,7	139,6	±	1,5	139,5	±	1,7	139,6	±	2,1	139,7	±	1,9	0,943
Serum potassium (mEq/L)	4,30	±	0,43	4,13	±	0,37[a]	4,08	±	0,34[a]	4,05	±	0,27[a]	4,16	±	0,29[d]	**0,001**
LDL (mg/dl)	122	±	27	120	±	28	116	±	23	115	±	23[a]	109	±	27[a,b]	**0,005**
HDL (mg/dl)	48	±	12	48	±	12	47	±	13	48	±	12	49	±	13	0,342
Total cholesterol (mg/dl)	196	±	32	197	±	30	190	±	26	185	±	23[a,b]	184	±	28[a,b]	**0,005**
Triglycerides (mg/dl)	127	±	70	144	±	97	141	±	105	119	±	80	122	±	75	0,072
GOT (U/L)	20,7	±	8,1	26,4	±	28,7	20,9	±	6,7	20,4	±	6,9	21,2	±	6,7	0,225
GPT (U/L)	23	±	14	30	±	22[a]	26	±	18	24	±	15[b]	25	±	19	**0,037**
GGT (U/L)	31	±	21	32	±	21	32	±	20	30	±	17	33	±	25	0,552
Alkaline phosphatase (U/L)	75	±	29	73	±	28	71	±	28	69	±	26[a,b]	69	±	26[a,b]	**0,001**
PTH (pg/ml)	45	±	17	47	±	19	47	±	17	49	±	20	54	±	25[a,b,c]	**0,003**
Calcitriol 1.25 D (ng/ml)	60	±	29	56	±	27	54	±	26	47	±	14				-
25 hydroxyvitamin D (ng/ml)	29	±	13	27	±	10	28	±	11	27	±	13	30	±	11	0,217
β-crosslaps (ng/ml)	0,46	±	0,22	0,46	±	0,24	0,43	±	0,22	0,43	±	0,22	0,44	±	0,22	0,376
Systolic BP (mmHg)	130,2	±	8,3	128,2	±	6,3	126,6	±	5,9[a]	125,4	±	4,5[a,b]	123,9	±	3,5[a,b,c]	**<0,001**
Diastolic BP (mmHg)	66,7	±	4,6	65,4	±	4,2[a]	64,8	±	4,1[a]	64,1	±	3,8[a]	62,8	±	3,8[a,b,c]	**<0,001**

Table S2. *Intragroup comparisons of patients treated with hydrochlorothiazide. a: p<0.05 vs corresponding pretreatment value; b: p<0.05 vs corresponding 3-month value; c: p<0.05 vs corresponding 6-month value; d: p<0.05 vs corresponding 12-month value. BP: blood pressure.*

	PRE				3 months				6 months				12 months				18 months				ANOVA for repeated measures
	INDAPAMIDE		THIAZIDE		INDAPAMIDE		THIAZIDE		INDAPAMIDE		THIAZIDE		INDAPAMIDE		THIAZIDE		INDAPAMIDE		THIAZIDE		
	Mean	± SD	Mean	± SD	Mean	± SD	Mean	± SD	Mean	± SD	Mean	± SD	Mean	± SD	Mean	± SD	Mean	± SD	Mean	± SD	*p*
2h Urine volume (ml)	87	± 30	75	± 28	80	± 36*	66	± 23*	78	± 42	61	± 29	79	± 20*	64	± 25*	82	± 29*	67	± 19*	**0,001**
2h Urine calcium (mg/L)	18,3	± 7,7	16,1	± 6,9	15,2	± 9,4	17,6	± 9,9	17,4	± 18,9	15,8	± 8,4	14,8	± 9,2	15,9	± 9,4	15,0	± 8,9	15,6	± 8,5	0,975
2h Urine citrate(mg/L)	388	± 200	482	± 290	473	± 270	623	± 435	456	± 245*	683	± 417*	438	± 226*	656	± 406*	431	± 219*	635	± 358*	**0,003**
2h Urine creatinine (mg/dl)	115	± 37	175	± 338	136	± 61*	164	± 56*	154	± 88*	179	± 77*	128	± 58*	162	± 60*	127	± 55	153	± 63	**0,025**
2h Urine pH	5,8	± 0,5	6,0	± 0,7	6,0	± 0,7	5,9	± 0,6	6,0	± 0,7	5,9	± 0,6	6,0	± 0,7	6,1	± 0,7	6,0	± 0,7	5,9	± 0,7	0,988
24h Urine volume (ml)	1999	± 578	2146	± 765	1941	± 605	2211	± 799	1983	± 682	2071	± 712	1899	± 702	2086	± 796	1958	± 671	2000	± 635	0,254
24h Urine calcium (mg/24h)	357	± 82	333	± 61	271	± 122	235	± 90	230	± 107	236	± 84	241	± 115	232	± 86	214	± 66	214	± 58	0,368
24h Urine citrate (mg/24h)	772	± 914	725	± 352	664	± 436	638	± 378	647	± 339	678	± 345	605	± 252	666	± 364	746	± 368	722	± 372	0,874
24hUrine creatinine (mg/24h)	1610	± 425	1567	± 483	1534	± 568	1411	± 435	1490	± 475	1415	± 399	1496	± 482	1453	± 418	1493	± 506	1422	± 364	0,435
24hUrinePhosphte (mg/24h)	1062	± 241	1031	± 289	996	± 342	862	± 286	974	± 302	895	± 315	962	± 316	949	± 272	985	± 317	943	± 273	0,223
24hUrineMagnesiu(mg/24h)	132	± 48	130	± 55	128	± 50	116	± 41	116	± 41	117	± 37	123	± 60	111	± 38	121	± 43	116	± 38	0,478
24h Urine oxalate (mg/24h)	34	± 18	34	± 15	28	± 13	35	± 22	27	± 12	30	± 11	31	± 11	34	± 6	37	± 19	24	± 15	0,882
24h Urine urate (mg/24h)	702	± 199	646	± 201	683	± 218	590	± 178	660	± 209	573	± 161	680	± 191	616	± 179	653	± 174	634	± 195	0,068
Serum glucose (mg/dl)	102	± 22	103	± 23	103	± 23	108	± 41	106	± 36	107	± 49	101	± 32	101	± 28	100	± 27	101	± 33	0,815
Serum urea (mg/dl)	35,0	± 6,8*	30,5	± 6,6*	35,9	± 7,9*	31,8	± 7,4*	34,3	± 7,2	31,4	± 7,4	34,2	± 8,0	32,7	± 8,8	33,9	± 8,9	30,8	± 7,8	**0,023**
Serum creatinine (mg/dl)	0,85	± 0,19	0,84	± 0,19	0,84	± 0,17	0,87	± 0,18	0,86	± 0,18	0,87	± 0,18	0,85	± 0,17	0,86	± 0,19	0,82	± 0,15	0,82	± 0,17	0,319
Serum urate (mg/dl)	5,3	± 1,2	5,4	± 1,3	5,7	± 1,2	6,0	± 1,4	5,7	± 1,3	5,7	± 1,2	5,8	± 1,2	5,9	± 1,4	5,6	± 1,3	5,8	± 1,3	0,618
Serum calcium (mg/dl)	9,52	± 0,40	9,51	± 0,44	9,48	± 0,39	9,50	± 0,47	9,47	± 0,38	9,44	± 0,44	9,48	± 0,38	9,35	± 0,44	9,41	± 0,41	9,38	± 0,41	0,600
Serum phosphate (mg/dl)	3,47	± 0,57	3,25	± 0,47	3,25	± 0,55	3,25	± 0,68	3,15	± 0,57	3,13	± 0,67	3,15	± 0,51	3,09	± 0,61	3,19	± 0,56	3,22	± 0,62	0,676
Serum magnesium (mg/dl)	2,03	± 0,18	1,95	± 0,13	1,97	± 0,12	1,94	± 0,16	1,97	± 0,15	1,96	± 0,20	1,96	± 0,16	1,95	± 0,17	1,95	± 0,16	1,95	± 0,16	0,346
Serum sodium (mEq/L)	139,8	± 2,0	139,7	± 1,7	139,8	± 1,9	139,6	± 1,5	139,1	± 2,3	139,5	± 1,7	139,0	± 2,1	139,6	± 2,1	139,9	± 1,9	139,7	± 1,9	0,755
Serum potassium (mEq/L)	4,22	± 0,40	4,30	± 0,43	4,93	± 0,79	4,13	± 0,37	4,09	± 0,40	4,08	± 0,34	4,04	± 0,39	4,05	± 0,27	4,11	± 0,30	4,16	± 0,29	0,478
LDL (mg/dl)	135	± 40	122	± 27	130	± 31	120	± 28	133	± 34*	116	± 23*	131	± 34*	115	± 23*	123	± 37	109	± 27	**0,018**
HDL (mg/dl)	50	± 10	48	± 12	51	± 10	48	± 12	51	± 12	47	± 13	52	± 11	48	± 12	51	± 9	49	± 13	0,212
Total cholesterol (mg/dl)	208	± 48	196	± 32	200	± 41	197	± 30	210	± 48*	190	± 26*	213	± 38*	185	± 23*	200	± 44	184	± 28	**0,023**
Triglycerides (mg/dl)	119	± 58	127	± 70	118	± 60	144	± 97	121	± 66	141	± 105	154	± 170	119	± 80	132	± 133	122	± 75	0,933

	PRE						3 months						6 months						12 months						18 months						ANOVA for repeated measures
	INDAPAMIDE			THIAZIDE			INDAPAMIDE			THIAZIDE			INDAPAMIDE			THIAZIDE			INDAPAMIDE			THIAZIDE			INDAPAMIDE			THIAZIDE			
	Mean		SD	Mean		SD	Mean		SD	Mean		SD	Mean		SD	Mean		SD	Mean		SD	Mean		SD	Mean		SD	Mean		SD	
		±			±			±			±			±			±			±			±			±			±		*p*
GOT (U/L)	20,6	±	5,4	20,7	±	8,1	21,3	±	7,0	26,4	±	28,7	20,1	±	4,8	20,9	±	6,7	20,7	±	6,5	20,4	±	6,9	20,1	±	4,6	21,2	±	6,7	0,332
GPT (U/L)	20	±	9	23	±	14	22	±	14	30	±	22	21	±	9	26	±	18	21	±	10	24	±	15	22	±	9	25	±	19	0,128
GGT (U/L)	29	±	24	31	±	21	29	±	28	32	±	21	27	±	19	32	±	20	26	±	12	30	±	17	28	±	17	33	±	25	0,353
Alkaline phosphatase (U/L)	74	±	23	75	±	29	73	±	24	73	±	28	72	±	23	71	±	28	73	±	24	69	±	26	73	±	23	69	±	26	0,825
PTH (pg/ml)	46	±	19	45	±	17	50	±	23	47	±	19	45	±	16	47	±	17	48	±	19	49	±	20	50	±	22	54	±	25	0,894
Calcitriol 1.25 D (ng/ml)	52	±	14	60	±	29	55	±	18	56	±	27	56	±	11	54	±	26	56	±	12	47	±	14	54	±	9				-
25hydroxyvitaminaD (ng/ml)	28	±	11	29	±	13	29	±	12	27	±	10	29	±	11	28	±	11	29	±	14	27	±	13	28	±	10	30	±	11	0,793
β-crosslaps (ng/ml)	0,47	±	0,21	0,46	±	0,22	0,44	±	0,21	0,46	±	0,24	0,46	±	0,23	0,43	±	0,22	0,44	±	0,21	0,43	±	0,22	0,41	±	0,20	0,44	±	0,22	0,936
Systolic BP (mmHg)	127,6	±	7,6	130,2	±	8,3	127,2	±	6,4	128,2	±	6,3	126,3	±	5,6	126,6	±	5,9	125,3	±	3,7	125,4	±	4,5	124,7	±	4,1	123,9	±	3,5	0,452
Diastolic BP (mmHg)	67,0	±	5,9	66,7	±	4,6	65,9	±	5,6	65,4	±	4,2	65,1	±	4,4	64,8	±	4,1	63,6	±	4,4	64,1	±	3,8	63,2	±	3,5	62,8	±	3,8	0,785

Table S3. Intergroup comparisons of patients treated with indapamide vs hydrochlorothiazide. *: months in which the difference between treatment groups was <0.05. BP: blood pressure.
